# Are “mystical experiences” essential for antidepressant actions of ketamine and the classic psychedelics?

**DOI:** 10.1007/s00406-024-01770-7

**Published:** 2024-02-27

**Authors:** Kenji Hashimoto

**Affiliations:** Division of Clinical Neuroscience, Chiba University Center for Forensic Mental Health, 1-8-1 Inohana, Chiba, 260-8670 Japan

**Keywords:** Arketamine, Depression, Dissociation, Esketamine, Hallucination, Ketamine, Psilocybin, Psychedelics

## Abstract

The growing interest in the rapid and sustained antidepressant effects of the dissociative anesthetic ketamine and classic psychedelics, such as psilocybin, is remarkable. However, both ketamine and psychedelics are known to induce acute mystical experiences; ketamine can cause dissociative symptoms such as out-of-body experience, while psychedelics typically bring about hallucinogenic experiences, like a profound sense of unity with the universe or nature. The role of these mystical experiences in enhancing the antidepressant outcomes for patients with depression is currently an area of ongoing investigation and debate. Clinical studies have shown that the dissociative symptoms following the administration of ketamine or (*S*)-ketamine (esketamine) are not directly linked to their antidepressant properties. In contrast, the antidepressant potential of (*R*)-ketamine (arketamine), thought to lack dissociative side effects, has yet to be conclusively proven in large-scale clinical trials. Moreover, although the activation of the serotonin 5-HT_2A_ receptor is crucial for the hallucinogenic effects of psychedelics in humans, its precise role in their antidepressant action is still under discussion. This article explores the importance of mystical experiences in enhancing the antidepressant efficacy of both ketamine and classic psychedelics.

## Introduction

Major depressive disorder (MDD), one of the most common psychiatric disorders, is characterized by persistent low mood (heightened negative emotions) or anhedonia (diminished positive emotions). Currently, treatments for depression include selective serotonin reuptake inhibitors, serotonin and norepinephrine reuptake inhibitors, and other antidepressants. However, significant concerns exist regarding these antidepressants. They are effective for approximately one-third of MDD patients, leaving a substantial portion exhibiting treatment-resistant depression (TRD) [1, 2]. Additionally, these medications often require several weeks to manifest their full effects, which can be particularly problematic for individuals with severe depression or suicidal thoughts. Furthermore, antidepressants can cause various side effects such as gastrointestinal (GI) issues (e.g., nausea, vomiting, or diarrhea), weight gain, sexual dysfunction, sleep disturbances, and emotional blunting [3–5]. These side effects frequently lead to poor adherence and discontinuation of treatment. Consequently, there is a critical unmet medical need to develop new antidepressants that can rapidly alleviate depressive symptoms, including in patients with TRD [6, 7].

**Fig. 1:**
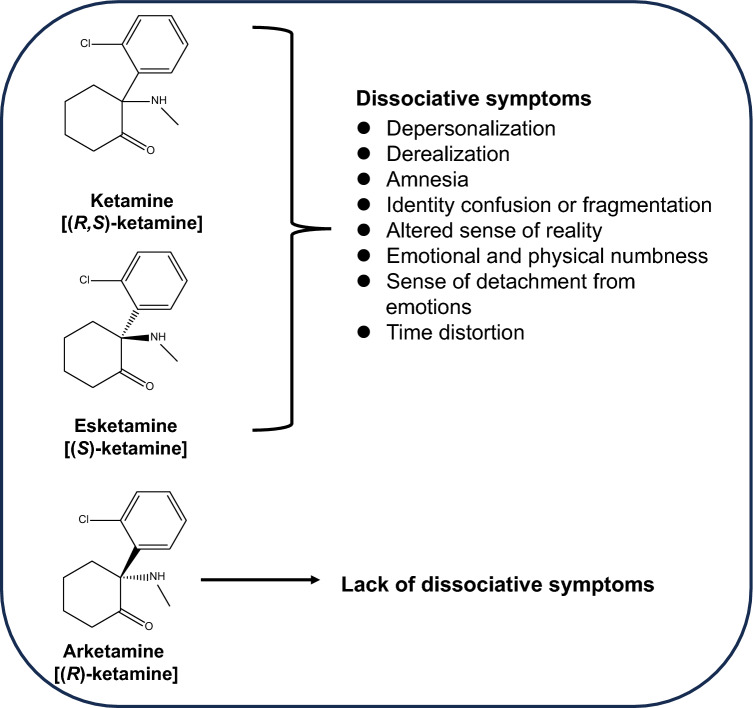
Chemical structures and dissociative symptoms of ketamine and its enantiomers. The figure illustrates the chemical structures of ketamine (racemic mixture), along with its two enantiomers: esketamine and arketamine. Clinical studies indicate that both ketamine and esketamine can induce dissociative symptoms in healthy volunteers and patients with MDD or BD. Conversely, arketamine is less likely to provoke dissociative symptoms at therapeutic doses. The potential mechanism underlying these dissociative symptoms is attributed to the inhibition of NMDAR by ketamine and esketamine

Ketamine (Fig. 1), originally known for its use as an anesthetic, has emerged as a significant treatment option for depression, particularly for cases with TRD including MDD and bipolar disorder (BD) [8–20]. One of the most notable features of ketamine in treating depression is its rapid onset of action. Unlike current antidepressants that can take weeks to show effects, ketamine can produce noticeable improvements in mood within hours or days. In addition, ketamine has been found to be particularly effective in individuals with TRD [21–24]. A recent study demonstrated that intravenous ketamine infusion is non-inferior to electroconvulsive therapy (ECT) as therapy for TRD without psychosis [25]. Furthermore, a comprehensive meta-analysis revealed that ECT is not superior to ketamine in the treatment of TRD, and that ketamine showed a significant rapid antidepressant effect over ECT [26]. Consequently, it is recommended that ketamine should be considered on par with ECT for the short-term management of depressive symptoms in outpatients with TRD [27]. Nonetheless, it is important to note that ketamine can cause side effects including dissociation, hallucinations, dizziness, elevated blood pressure during administration, and the potential for abuse with repeated use [8, 28].

**Fig. 2:**
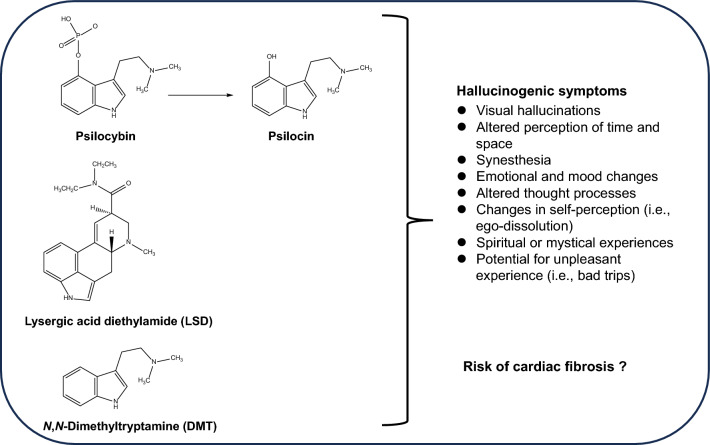
Chemical structures of classic psychedelics, their hallucinogenic symptoms, and risk of cardiac fibrosis. This figure displays the chemical structures of classic psychedelics, including psilocybin and its primary metabolite psilocin, lysergic acid diethylamide (LSD), and *N*,*N*-dimethyltryptamine (DMT). Psilocybin, found in magic mushrooms, is metabolized into the pharmacologically active compound psilocin. LSD, a synthetic psychedelic, was developed at Sandoz laboratories in Switzerland. DMT, structurally similar to 5-hydroxytryptamine (5-HT: serotonin), occurs naturally in various plants and animals, including humans. In South American indigenous cultures, it is traditionally ingested in the form of ayahuasca, a ceremonial spiritual medicine. These serotonergic classic psychedelics, known for inducing mystical experiences, primarily exert their effects through the activation of the 5-HT_2A_ receptor. Stimulation of the 5-HT_2B_ receptor by psilocin and LSD may potentially increase the risk of cardiac fibrosis [143]

The use of classic psychedelics such as psilocybin (4-phosphoryloxy-*N*,*N*-dimethyltryptamine) (found in magic mushrooms), lysergic acid diethylamide (LSD), and *N,N*-dimethyltryptamine (DMT) (found in ayahuasca) (Fig. 2) in treating severe depression represents a growing area of interest in psychiatric research [29–34]. Like ketamine, psychedelics such as psilocybin can produce rapid and sustained antidepressant actions in TRD patients, including MDD and BD [35–39]. It is also currently unclear whether mystical experiences induced by psychedelics are associated with their antidepressant actions in patients with MDD [40–42].

In this review, the author explores the relationships between the robust antidepressant effects of ketamine and psychedelics and the mystical experiences that accompany their application in the treatment of depression.

## Dissociative and antidepressant effects of ketamine and its enantiomers

### Preclinical studies

Ketamine is a racemic mixture of (*R*)-ketamine (arketamine) and (*S*)-ketamine (esketamine) (Fig. 1). Esketamine shows a higher affinity for the *N*-methyl-D-aspartate receptor (NMDAR) than arketamine. Despite having a lower NMDAR affinity, arketamine demonstrates more potent and sustained antidepressant-like effects in various animal models of depression [43–50]. Additionally, compared to ketamine and esketamine, arketamine’s side effects, such as hyperlocomotion, prepulse inhibition, and abuse liability, are less severe in rodents and monkeys [44, 47, 51–53]. Thus, arketamine may emerge as a novel antidepressant with fewer side effects than ketamine and esketamine.

Non-competitive NMDAR antagonists such as phencyclidine (PCP) and ketamine are known for inducing dissociative symptoms in humans. These symptoms include altered perceptions of time, space, and the environment, leading to feelings of disconnection from surroundings and distorted spatial awareness. A notable dissociative effect is a sense of detachment or estrangement from oneself, sometimes culminating in an out-of-body experience [8, 54, 55] (Fig. 1). However, the precise molecular and cellular mechanisms behind ketamine-induced dissociation are not fully understood.

Assessing dissociative symptoms in humans relies heavily on self-reporting of mental states, making it challenging to replicate these conditions for behavioral tests in rodents [56]. In 2020, Vesuna and colleagues [57] reported a significant discovery: oscillation rhythms in layer 5 neurons of the retrosplenial cortex are crucial for dissociation-like experiences induced by ketamine, PCP, or dizocilpine. In contrast, memantine, a low-affinity non-competitive NMDAR antagonist that does not cause dissociation in humans, did not induce these oscillation rhythms. They also performed behavioral tests on mice to assess dissociation-like symptoms. Their findings suggest that 1–3 Hz oscillation rhythm in the retrosplenial cortex is essential for inducing dissociation-like behaviors in mice due to ketamine [57], though further detailed research is required.

In 1989, Olney and colleagues [58] demonstrated that NMDAR antagonists like PCP, ketamine, and dizocilpine induce neuropathological changes (specifically, neuronal vacuolization) in the rat brain’s retrosplenial cortex. The severity of these changes correlated with each compound’s potency at the NMDAR. Dizocilpine and ketamine, as well as esketamine, significantly induced the expression of heat shock protein HSP-70, a marker for neuronal injury, in the retrosplenial cortex of the rat brain. In contrast, arketamine did not trigger HSP-70 expression in this region [59]. The neuropathological alterations observed in the retrosplenial cortex following PCP, ketamine, and esketamine injections might be linked to their dissociative side effects. Future studies, particularly focusing on the two enantiomers of ketamine, would be invaluable in confirming the role of NMDAR in the induction of dissociation-like behaviors and oscillation rhythms in the layer 5 neurons of the retrosplenial cortex [56].

### Clinical studies

#### Dissociative symptoms and antidepressant effects after injection of ketamine or esketamine

In 1997, Vollenweider and colleagues [60] found that increases in glucose utilization in the frontal and left temporal cortex, induced by esketamine, were correlated with ego disintegration and hallucinations in healthy volunteers. In contrast, equivalent doses of arketamine reduced glucose utilization in various brain regions. Notably, arketamine did not trigger psychotic or dissociative symptoms but rather induced relaxation. This study indicates that the esketamine-induced metabolic hyperactivity in the frontal areas resembles the metabolic alterations observed in acute psychotic episodes in patients with schizophrenia. Additionally, it suggests that arketamine is unlikely to cause schizophrenia-like symptoms in healthy individuals.

In 2014, Luckenbaugh and colleagues [61] reported a significant link between ketamine-induced dissociative side effects and its antidepressant efficacy in patients with TRD (*n* = 108). They found a notable association between increased scores on the Clinician-Administered Dissociative States Scale (CADSS) at 40 min and the improvement in the Hamilton Depression Rating Scale (HDRS) scores at 230 min and day 7 following ketamine administration. However, they observed no correlation between changes in the Young Mania Rating Scale (YMRS) or the Brief Psychiatric Rating Scale (BPRS) positive symptom scores at 40 min and HDRS improvement at any time point with ketamine.

In 2018, Niciu and colleagues [62] reported that shifts in depersonalization items on the CADSS, indicative of dissociative side effects, were correlated with changes in depressive symptoms. Additionally, a 2019 study, which analyzed YouTube videos of depressed patients receiving ketamine infusions, suggested that a self-reported feeling of lightness or floating was linked to relief from depressive symptoms [63]. Conversely, Wilkinson and colleagues [64] found no correlation between CADSS scores and the antidepressant effects of ketamine in TRD patients (*n* = 54). These findings highlight the inconsistent nature of the relationship between ketamine-induced dissociation and its antidepressant effects.

An analysis using three trials showed that the antidepressant effects of ketamine in TRD patients (38 with MDD and 44 with BD) are not mediated by the dissociative depersonalization subtype symptom of floating [65]. In a subsequent study, Ballard and Zarate [66] concluded that dissociation is not a necessary component for the antidepressant actions of ketamine. Moreover, a systematic review encompassing 21 studies revealed that total score for ketamine-induced CADSS does not consistently correlate with its antidepressant outcomes [67]. Overall, it appears that ketamine-induced dissociation is not essential for its antidepressant actions, though additional research is required to fully comprehend the relationship between dissociation and the antidepressant effects of ketamine.

In 2019, esketamine nasal spray was approved for TRD in the United State (US) of America and Europe, despite concerns regarding its efficacy and Food Drug Administration (FDA) approval [68]. A recent study utilizing data from the US Food and Drug Adverse Event Reporting System (FAERS) highlighted potential adverse effects and risks associated with the clinical use of esketamine, particularly focusing on its long-term effectiveness, potential for addiction, and suicidal risks [69]. It found the most significant indications for dissociation, dissociative disorder, and sedation. Alarmingly, the frequencies of suicidal ideation and attempt were relatively high, underscoring the need for caution when using esketamine in clinical settings [69]. Therefore, while esketamine nasal spray offers rapid antidepressant benefits, it also introduces various adverse effects and potential hazards.

Meta-analyses revealed that the antidepressant effectiveness of esketamine nasal spray is less pronounced compared to intravenous ketamine injections [70, 71]. The reasons for this difference remain uncertain, but aspects such as the lower bioavailability of the nasal spray [9] and the lack of the arketamine enantiomer in the esketamine formulation could be contributing factors. A comparative study of esketamine and arketamine in MDD patients is crucial to determine which enantiomer significantly contributes to ketamine’s antidepressant effects. In 2022, Chen and colleagues [72] reported that the antidepressant effects of esketamine nasal spray in TRD patients were not correlated with dissociative symptoms. In the TRANSFORM-2 study, the response rate at day 2 and day 28 was similar regardless of whether patients experienced significant dissociation following the first dose. Moreover, dissociation scores did not influence the reduction in depression score at day 2 or 28 in TRANSFORM-2, nor did they affect the time to depression relapse in the SUSTAIN-1 trial. This evidence from two phase 3 trials indicates that the antidepressant effects of nasal esketamine spray do not dependent on its dissociative symptoms [72].

Taken together, these findings suggest that the antidepressant effects of both ketamine and esketamine are independent of their dissociative symptoms. Considering the role of NMDAR inhibition in the side effects (such as dissociative symptoms) of NMDAR antagonists [73, 74], it seems unlikely that NMDAR plays a crucial role in the antidepressant effects of ketamine [9–11, 13–17, 75].

#### Arketamine

Arketamine is known for its greater and more enduring antidepressant-like effects in various animal models of depression. However, there are relatively few studies exploring its antidepressant effects in TRD patients. A pioneering open-label pilot study in Brazil revealed that a single intravenous injection of arketamine (0.5 mg/kg, 40 min) elicited rapid and sustained antidepressant effects in a small group of female TRD patients (*n* = 7) [76]. In contrast, a subsequent placebo-controlled pilot study in Brazil indicated that arketamine did not significantly outperform a placebo in TRD patients (*n* = 10) [77]. Additionally, the same research team in Brazil reported that arketamine at doses at 0.5 and 1.0 mg/kg had rapid-acting antidepressant effects in a small cohort of patients (*n* = 6) with bipolar depression [78]. Across these three studies, the reported side effects (i.e., dissociation) of arketamine were very low [76–78].

In 2021, Perception Neuroscience, based in New York, US, released data of a phase 1 single ascending dose study of PCN-101 (arketamine) involving healthy adult volunteers [79]. The study found that intravenous arketamine was safe and well tolerated at all tested doses, up to 150 mg, and there were no serious adverse events reported. Notably, it was observed that significantly higher doses of arketamine were required to induce perceptional changes (a type of dissociation side effect) compared to esketamine.

In January 2023, a press release reported that the phase 2a trial of PCN-101 (arketamine) in TRD patients did not achieve statistical significance on the primary endpoint [79]. However, in June 2023, further analysis from the phase 2a trial data revealed differences between the US and Europe cohorts. Specifically, the US subgroup showed clinically meaningful improvement in depression scores for up to two weeks following a single intravenous infusion of arketamine (60 mg, 40 min). Importantly, arketamine was generally well tolerated in this trial, with no serious adverse events and an acceptable safety profile. There were no significant differences in sedation and dissociative symptoms between the arketamine and placebo groups. Overall, these findings suggest that arketamine does not produce dissociative side effects in humans at doses effective for treating depression. To further investigate the role of dissociative symptoms in ketamine’s antidepressant effects, conducting a double-blind, randomized controlled trial is necessary. This study would compare the effects of arketamine with a placebo (or esketamine) in TRD patients with MDD or BD.

## Hallucinogenic and antidepressant effects of classic psychedelics

### Findings from preclinical studies

Psychedelics such as psilocybin primarily act on the 5-hydroxytryptamine (5-HT) 5-HT_2A_ receptors in the brain, leading to alterations in perception, thought, and mood [80]. Head-twitch response (HTR) is a rapid, rotational head movement observed in rodents after administration of 5-HT_2A_ receptor agonists such as psilocybin and LSD. The potency of psychedelics determined via mouse HTR is highly correlated with potencies to elicit hallucinations in humans [81]. Furthermore, HTR induced by psychedelics in mice could be blocked by potent 5-HT_2A_ receptor antagonists or deletion of the 5-HT_2A_ receptor gene [82–85]. Thus, it is likely that 5-HT_2A_ receptor could play a role in HTR induced after administration of psychedelics. A recent study showed that the 5-HT_1A_ receptor agonist 8-OH DPAT attenuated psilocybin-induced HTR in mice [86], suggesting inhibitory effects of 5-HT_1A_ receptor for the HTR caused by psychedelics. Furthermore, unlike esketamine and the selective NMDAR antagonist dizocilpine, psilocybin did not induce HSP-70 expression in the rat retrosplenial cortex [87], indicating that psilocybin could be a safer option for clinical applications in comparison to esketamine.

In terms of potential antidepressant-like effects of psilocybin, the data are mixed regarding the role of 5-HT_2A_ receptor activation. This uncertainty arises in part from research utilizing rodents that lack depression-like behaviors, potentially limiting accurate predictions about the clinical effectiveness of antidepressant candidates [88–90]. One study showed that 5-HT_2A_ receptor antagonist ketanserin did not block antidepressant-like effects of psilocybin in chronically stressed male mice with depression-like behaviors [91], suggesting that altered perception may not be necessary for its antidepressant actions.

It is currently unclear if 5-HT_2A_ receptor can mediate antidepressant effects of psychedelics such as psilocybin, because several non-hallucinogenic analogs of psychedelics with antidepressant-like properties have been developed [92–94]. In 2023, Qu and colleagues [95] compared the effects of DOI (2,5-dimethoxy-4-iodoamphetamine: a hallucinogenic psychedelic drug with potent 5-HT_2A_ receptor agonism), lisuride (non-hallucinogenic psychedelic analog with 5-HT_2A_ and 5-HT_1A_ receptor agonisms), and arketamine on depression-like behavior and the decreased dendritic spine density in the brain of lipopolysaccharide (LPS)-treated mice. Both lisuride and arketamine ameliorated the increased immobility time of forced swimming test (FST), and the decreased dendritic spine density in the medial prefrontal cortex (PFC) and hippocampus of LPS-treated mice. In contrast, DOI did not improve these changes of LPS-treated mice. This study suggests that 5-HT_2A_ receptor may not play a major role in rapid-acting antidepressant actions of psychedelics although further detailed studies is needed. In addition, it is likely that potent 5-HT_1A_ receptor agonism of lisuride plays a role in a lack of HTR in rodents [86, 95]. Unfortunately, the effects of psilocybin with antidepressant effects in depressed patients were not investigated in this study [95].

Subsequently, Liu and colleagues [96] reported that pretreatment (6 days before LPS) with arketamine, but not DOI and lisuride, ameliorated body weight loss, splenomegaly, the increased immobility time of FST, and the decreased expression of synaptic protein in the PFC of LPS-treated mice. Furthermore, pretreatment with arketamine, but not DOI and lisuride, significantly ameliorated the increased FST immobility time, the reduced sucrose preference in the sucrose preference test, and the decreased expression of synaptic protein in the PFC of CRS (chronic restrain stress)-exposed mice. Unlike to arketamine, both DOI and lisuride do not exhibit long-lasting prophylactic effects in mouse models of depression.

A recent study demonstrated that LSD and psilocin, the primary metabolite of psilocybin, bind directly to TrkB (a receptor for brain-derived neurotrophic factor [BDNF]), exhibiting affinities that are 1,000-fold higher than those of other antidepressants [97]. Furthermore, the study revealed that psychedelics and antidepressants bind to distinct, yet partially overlapping, sites within the transmembrane domain of TrkB dimers [97]. Given the established importance of BDNF–TrkB signaling in the rapid and sustained antidepressant-like effects observed with ketamine and arketamine [44, 46, 49, 98–101], these findings are particularly intriguing. They suggest that high-affinity TrkB positive allosteric modulators, which do not activate the 5-HT_2A_ receptor, may maintain the antidepressant potential of psychedelics without inducing hallucinogenic effects [102]. Consequently, there is an ongoing debate over the role of 5-HT_2A_ receptor in the antidepressant actions of psychedelics such as psilocybin.

### Findings from clinical studies

The unique subjective experiences induced by psychedelics like psilocybin are characterized by phenomena such as ego dissolution (loss of self-awareness), indescribable insights, and a profound sense of unity and connection with others (Fig. 2) [36, 103–105]. Psilocybin, upon ingestion, is converted by the body into psilocin (4-hydroxy-*N,N*-dimethyltryptamine: 4-hydroxy DMT), the pharmacologically active compound, which predominantly binds to the 5-HT_2A_ receptor (Fig. 2).

The hallucinogenic effects of psychedelics like psilocybin and LSD in health volunteers are known to be mediated by the 5-HT_2A_ receptor, as evidenced by the fact that these effects can be inhibited by the 5-HT_2A_ receptor antagonist ketanserin [106–108]. A study employing positron emission tomography (PET) revealed a strong correlation between the intensity of psychedelic experiences, 5-HT_2A_ receptor occupancy, and plasma psilocin levels [109]. Thus, activation of the 5-HT_2A_ receptor is likely a contributing factor to the hallucinogenic effects of psychedelics in humans. Research showed that a strong of oceanic boundlessness, akin to mystical-type experiences, under psilocybin was predictive of an antidepressant response in TRD patients (*n* = 20) [110]. This suggests that the nature of the acute mystical experiences plays a crucial role in mediating the long-term antidepressant effects. However, it remains uncertain whether 5-HT_2A_ receptor activation also plays a role in the potential antidepressant actions of psychedelics.

In 2022, Gukasyan and colleagues [111] conducted a 12-month prospective follow-up study examining the effectiveness and safety of psilocybin-assisted therapy for patients with severe MDD (*n* = 24). Significant reductions from baseline in HAMD scores were noted at 1, 3, 6, and 12 months. At the 12-month mark, the treatment response (defined as a greater than 50% reduction in HAMD score from baseline) was 75%, and remission rate was 58%. Notably, participants’ reports of personal meaning, spiritual experiences, and mystical experiences following sessions were linked to enhanced well-being at 12 months, yet these did not correlate with improvement in depression. In a comprehensive naturalistic study, involving individuals (*n* = 302) who planned to undergo a psychedelic experience, several factors were found to significantly influence changes in depressive symptoms [112]. These factors included the individuals’ medicinal motivations, their history of previous psychedelic use, the dosage of the drug, and the nature of the acute psychedelic experience, particularly the occurrence of an emotional breakthrough [112]. Moreover, a placebo-controlled, double-blind, randomized trial revealed that the subjective effects experienced from a single dose of psilocybin in MDD patients (n = 52) were not associated with a reduction in depressive symptoms two weeks after treatment [113].

In a 2023 exploratory placebo-controlled study involving moderate to severe patients (*N* = 19) with MDD, improvements in depression and anxiety were observed following both placebo and psilocybin treatments, with no significant differences between the two groups [114]. The psilocybin treatment showed high response (66.7%) and remission rates (46.7%). However, the intensity of mystical experiences during psilocybin administration did not correlate with subsequent antidepressant effects [114]. Therefore, it appears unlikely that psilocybin-induced mystical experiences contribute to its antidepressant effects in MDD patients, although it is important to note the sample sizes of these studies.

In 2023, Rosenblat and colleagues [115] reported a groundbreaking case: an adult with TRD who received psilocybin therapy following premedication with trazodone, a potent 5-HT_2A_ receptor antagonist. This case raises the possibility that the antidepressant effects of psilocybin may not solely depend on 5-HT_2A_ receptor activation or its psychedelic properties. However, further research is necessary to fully understand the role of 5-HT_2A_ receptor activation and its psychedelic impact in antidepressant mechanism of psychedelics like psilocybin.

To delve deeper into the role of the 5-HT_2A_ receptor in psilocybin’s antidepressant effects, a comprehensive study design is proposed: a double-blind, randomized controlled trial comparing the effects of psilocybin with and without the 5-HT_2A_ receptor antagonists such as ketanserin and volinanserin (MDL 100,907). Additionally, a similar ongoing study (NCT05710327) is examining the effects of psilocybin (25 mg) combined with risperidone (1 mg), which blocks dopamine D_2_ and 5-HT_2A_ receptors, in TRD patients [116].

There are currently a limited number of studies indicating the antidepressant effects of LSD and DMT in patients with MDD. A randomized, placebo-controlled crossover study found that LSD treatment (200 μg across two sessions) significantly alleviated anxiety and depressive symptoms in patients (*n* = 42), some of whom had a life-threatening illness [117]. Notably, positive acute subjective drug effects and mystical-type experiences in the first session correlated with long-term reductions in anxiety symptoms [117]. Another randomized, placebo-controlled crossover study revealed that a low dose of LSD (26 μg) decreased depressive scores in depressed patients, albeit with various subjective effects [118]. Additionally, an open-label study reported that a single dose of ayahuasca produced rapid antidepressant effects in six inpatients with depression [119]. More recently, an open-label study demonstrated that DMT treatment (initially 0.1 mg/kg, followed by 0.3 mg/kg) lowered depressive scores in seven MDD patients, though it increased blood pressure, heart rate, anxiety, psychedelic, and psychotomimetic effects [120]. Given the scarcity of research, the extent to which mystical experiences induced by LSD or DMT contribute to their antidepressant effects in depressed patients remains uncertain.

Furthermore, recent studies have highlighted non-hallucinogenic compound lisuride, which shows antidepressant-like effects in preclinical models [95, 121]. A notable study from Japan demonstrated that lisuride maleate (0.075 mg/day over 12 weeks) improved depressive symptoms in patients suffering from post-stroke depression [122]. This study demonstrated that the antidepressant effects of lisuride in these patients do not have a rapid onset. Given lisuride’s established use in treating Parkinson’s disease, it is intriguing to consider its potential in treating MDD patients.

## Conclusion remarks and future directions

A wealth of clinical evidence suggests that the dissociative symptoms triggered by ketamine or esketamine might not be crucial for their antidepressant effects in patients with TRD, including MDD or BD. If large-scale clinical trials confirm the antidepressant efficacy of arketamine in TRD patients, this could indicate that ketamine-induced dissociation is not essential for its robust antidepressant effects. Currently, clinical trials investigating arketamine for depression are being conducted by several pharmaceutical companies, including Perception Neuroscience (USA), Otsuka (Japan), HengRui (China), and Nhwa (China) [15].

The precise role of 5-HT_2A_ receptor activation and its associated hallucinogenic effects in the antidepressant action of psychedelics remains unclear. A recent study has shown that psilocybin, unlike lisuride, can induce antidepressant-like effects in 5-HT_2A_ receptor knock-out mice subjected to repeated swimming stress (10 min daily for five consecutive days) [123]. This implies that the antidepressant-like effects of psilocybin might not involve the 5-HT_2A_ receptor. Furthermore, recent clinical studies suggest there is no significant relationship between the hallucinogenic and antidepressant effects of psilocybin [111, 113–115]. Investigating whether the antidepressant effects of psychedelics like psilocybin can be inhibited by treatment with 5-HT_2A_ receptor antagonist in depressed patients is a topic of significant interest. Furthermore, clinical trials investigating new non-hallucinogenic psychedelics for the treatment of depression are garnering significant interest [124–126].

The molecular mechanisms driving the potent antidepressant effects of serotonergic psychedelics are not yet fully understood. These classic psychedelics are known to primarily influence the serotonergic system in the brain, but they also exert significant effects on the GI tract. Given that GI tract is home to a vast array of 5-HT receptors, psychedelics can induce a range of effects, including altered gut motility. This can manifest as symptoms such as nausea, vomiting, diarrhea, or altered perception of GI sensations [127–129]. Recent studies demonstrated that the gut–brain axis via the vagus nerve plays a crucial role in the stress resilience of the serotonergic entactogen 3,4-methylenedixoymethamphetamine in rodents [130, 131]. Moreover, various studies propose that the gut–brain axis might also play a role in the antidepressant-like actions of ketamine and arketamine [132–137]. Considering that over 95% of the body’s 5-HT is found in the GI tract [138, 139], the role of 5-HT in this region and its subsequent impact on the gut–brain axis are important factors to consider in understanding the antidepressant mechanisms of ketamine and serotonergic psychedelics [140, 141].

Finally, the use of ketamine and psychedelics for depression therapy, while showing promise, raises several major concerns: First, ketamine and psychedelics can induce significant side effects, including psychotomimetic and dissociative symptoms, and hallucinogenic effects during the acute phase of the experience. The primary challenge in blind studies is preserving their blind aspect. Due to the distinct mystical experience often induced by ketamine and psychedelics, participants and researchers may easily deduce whether they have received the actual substance or a placebo. Long-term effects and the potential for psychological harm in vulnerable individuals are not fully understood. A recent study, analyzing data from the US FAERS, underscored potential negative outcomes and hazards (i.e., dissociation, sedation, suicidal ideation, suicidal attempt) linked to the clinical application of esketamine nasal spray [69]. Additionally, a recent longitudinal observational study, which included samples from adult populations in the US and UK (total 9,732 participants), revealed correlations between the use of psychedelic and the occurrence of unusual visual experiences that manifest after the acute pharmacological effects have diminished [142]. Recent research indicated that prolonged microdosing of psychedelics like psilocybin and LSD, over several months or more, may increase the risk of cardiac fibrosis [143]. This is attributed to the stimulation of the 5-HT_2B_ receptor by these substances, potentially leading to the development of fibrosis [143]. Second, ketamine and psychedelics can trigger or exacerbate psychotic episodes in individuals with a personal or family history of psychiatric disorders. A prior investigation demonstrated that the repeated administration of esketamine, as opposed to arketamine, augmented locomotor activity in mice following additional methamphetamine exposure [144], indicating an elevated likelihood of psychosis in subjects treated with esketamine. Third, both ketamine and psychedelics generally have a potential for addition. There is concern about their misuse outside a therapeutic context. A study, analyzing data from the US FAERS, highlighted the potential risk of abuse associated with the clinical use of esketamine nasal spray [69]. Fourth, the necessity of psychotherapy in conjunction with psychedelic treatment remains a question [145–148]. Finally, the off-label use of ketamine and psychedelics challenges current medical, ethical, and societal norms around drug use and mental health treatment, requiring careful consideration and potentially new frameworks for understanding and managing mental health.
